# Case Report: Training Monitoring and Performance Development of a Triathlete With Spinal Cord Injury and Chronic Myeloid Leukemia During a Paralympic Cycle

**DOI:** 10.3389/fresc.2022.867089

**Published:** 2022-06-30

**Authors:** Oliver J. Quittmann, Benjamin Lenatz, Patrick Bartsch, Frauke Lenatz, Tina Foitschik, Thomas Abel

**Affiliations:** ^1^Department IV: Movement Rehabilitation, Neuromechanics and Paralympic Sport, Institute of Movement and Neurosciences, German Sport University Cologne, Cologne, Germany; ^2^European Research Group in Disability Sport (ERGiDS), Bonn, Germany; ^3^Gesundheitszentrum Gektis, Radevormwald, Germany

**Keywords:** spinal cord injury (SCI), chronic myeloid leukemia (CML), case report, paratriathlon, training, TRIMP, sRPE, performance

## Abstract

**Introduction:**

Paratriathlon allows competition for athletes with various physical impairments. The wheelchair category stands out from other paratriathlon categories, since competing in swimming, handcycling, and wheelchair racing entails substantial demands on the upper extremity. Therefore, knowledge about exercise testing and training is needed to improve performance and avoid overuse injuries. We described the training monitoring and performance development throughout a Paralympic cycle of an elite triathlete with spinal cord injury (SCI) and a recent diagnosis of chronic myeloid leukemia (CML).

**Case Presentation/Methods:**

A 30-year-old wheelchair athlete with 10-years experience in wheelchair basketball contacted us for guidance regarding testing and training in paratriathlon. Laboratory and field tests were modified from protocols used for testing non-disabled athletes to examine their physical abilities. In handcycling, incremental tests were used to monitor performance development by means of lactate threshold (*P*_OBLA_) and define heart rate-based training zones. All-out sprint tests were applied to calculate maximal lactate accumulation rate (${\dot{\text{V}}\text{La}}_{\max}$) as a measure of glycolytic capabilities in all disciplines. From 2017 to 2020, training was monitored to quantify training load (TL) and training intensity distribution (TID).

**Results:**

From 2016 to 2019, the athlete was ranked within the top ten at the European and World Championships. From 2017 to 2019, annual TL increased from 414 to 604 h and demonstrated a shift in TID from 77-17-6% to 88-8-4%. In this period, *P*_OBLA_ increased from 101 to 158 W and ${\dot{\text{V}}\text{La}}_{\max}$ decreased from 0.56 to 0.36 mmol·l^−1^·s^−1^. TL was highest during training camps. In 2020, after he received his CML diagnosis, TL, TID, and *P*_OBLA_ were 317 h, 94-5-1%, and 108 W, respectively.

**Discussion:**

TL and TID demonstrated similar values when compared with previous studies in para-swimming and long-distance paratriathlon, respectively. In contrast, relative TL during training camps exceeded those described in the literature and was accompanied by physical stress. Increased volumes at low intensity are assumed to increase *P*_OBLA_ and decrease ${\dot{\text{V}}\text{La}}_{\max}$ over time. CML treatment and side effects drastically decreased TL, intensity, and performance, which ultimately hindered a qualification for Tokyo 2020/21. In conclusion, there is a need for careful training prescription and monitoring in wheelchair triathletes to improve performance and avoid non-functional overreaching.

## Introduction

Paratriathlon is an endurance sport for people with a physical impairment that made its Paralympic debut in 2016 and is increasingly featured in newspaper articles (1). The athletes compete in various sports classes covering the ambulant/standing, visually impaired, and wheelchair (PTWC) categories (2). Within the PTWC, there are two sports classes for most (PTWC1) and least (PTWC2) impaired wheelchair users. During the competition, the PTWC1 starts with a time advantage before PTWC2 athletes (3). Although the ambulant/standing and visually impaired categories appear to be rather similar to conventional triathlon, the locomotion in PTWC is substantially different. Since these athletes purely rely on their upper extremities, handcycling and wheelchair racing are used as equivalent to leg cycling and running, respectively.

Previous case studies already reported training characteristics in para-swimming (4), handcycling (5, 6), wheelchair racing (7), and long-distance amputee paratriathlon (8). Training characteristics and performance development of a paratriathlon long-distance world champion (with unilateral below-the-knee amputation) were described over a period of 19 months (8). Mean training volumes were found to be lower when compared with non-disabled Olympic-distance triathletes and attained values of 8 ± 3, 6 ± 4, and 2 ± 1 h/w in swimming, cycling, and running, respectively (8). However, training practices may have changed from 2011/2012 (when the data of this case study were recorded) to the present and do not necessarily apply to triathletes with spinal cord injury (SCI).

Traumatic SCIs are defined as damage to the spinal cord due to a mechanical trauma “that temporarily or permanently causes changes in its function” (9). Depending on the level and (in)completeness of the lesion, afferent and efferent neurons as well as autonomic function are affected to a certain extent. Although paraplegia indicates that two limbs are affected (predominantly damage to thoracic, lumbar, and sacral regions), tetraplegia refers to impairment in all four limbs. The incidence of traumatic SCI was found to differ among age groups (10) and regions and is ~1 case per 100,000 individuals in Germany (11). The major causes of traumatic SCIs are accidents in motor vehicles (11). As a treatment of traumatic SCIs, surgical decompression in an early state and neuroprotective and/or regenerative strategies in the follow-up may help to reduce symptoms and side effects (12). Besides, habitual exercise was highlighted as “an effective countermeasure for addressing physical deconditioning after SCI” (13). However, since wheelchair athletes purely rely on their upper extremities, a high prevalence of upper extremity injuries was highlighted (14).

Chronic myeloid leukemia (CML) is a myeloproliferative neoplasm that accounts for approximately 15% of newly diagnosed cases of leukemia in adults (15). The incidence of CML is stated to be 1–2 cases per 100,000 adults with a mortality of 1–2% (15). The genetic origin of CML is assumed to be a fusion oncogene (BCR-ABL1) on the so-called “*Philadelphia chromosome”* (22q11.2) (15). The first-line treatment of CML in the chronic phase is different types of tyrosine kinase inhibitors (TKIs) that lead to a normal life expectancy for most patients (16). It was recently shown that the majority of patients with CML receiving TKI therapy experience severe fatigue that causes an increased need for sleep, a reduction of physical activity, and consequently an impaired quality of life (17). However, alternative treatments to TKIs indicated promising results in terms of molecular response and side effects (18). Besides a stable deep molecular response, CML therapy aims for treatment-free remission (16). While moderate exercise was found to be a promising tool in the treatment of acute myeloid leukemia (19), findings regarding the interaction of CML and exercise are still lacking.

This case report addresses several research gaps. First, longitudinal studies on the training and development of Paralympic athletes are generally sparse—especially over several years. Second, evidence on how paratriathletes prepare for the Paralympic Games is lacking. Third, the PTWC category demonstrates the highest difference from conventional triathlon when compared with other categories and as such requires the implementation of modified tools in exercise testing and training. Finally, to the best of our knowledge, there is no study that analyzed the acute reaction to and side effects of CML and its treatment on performance, training, and wellbeing in a highly trained athlete.

## Case Description

**Figure 1:**
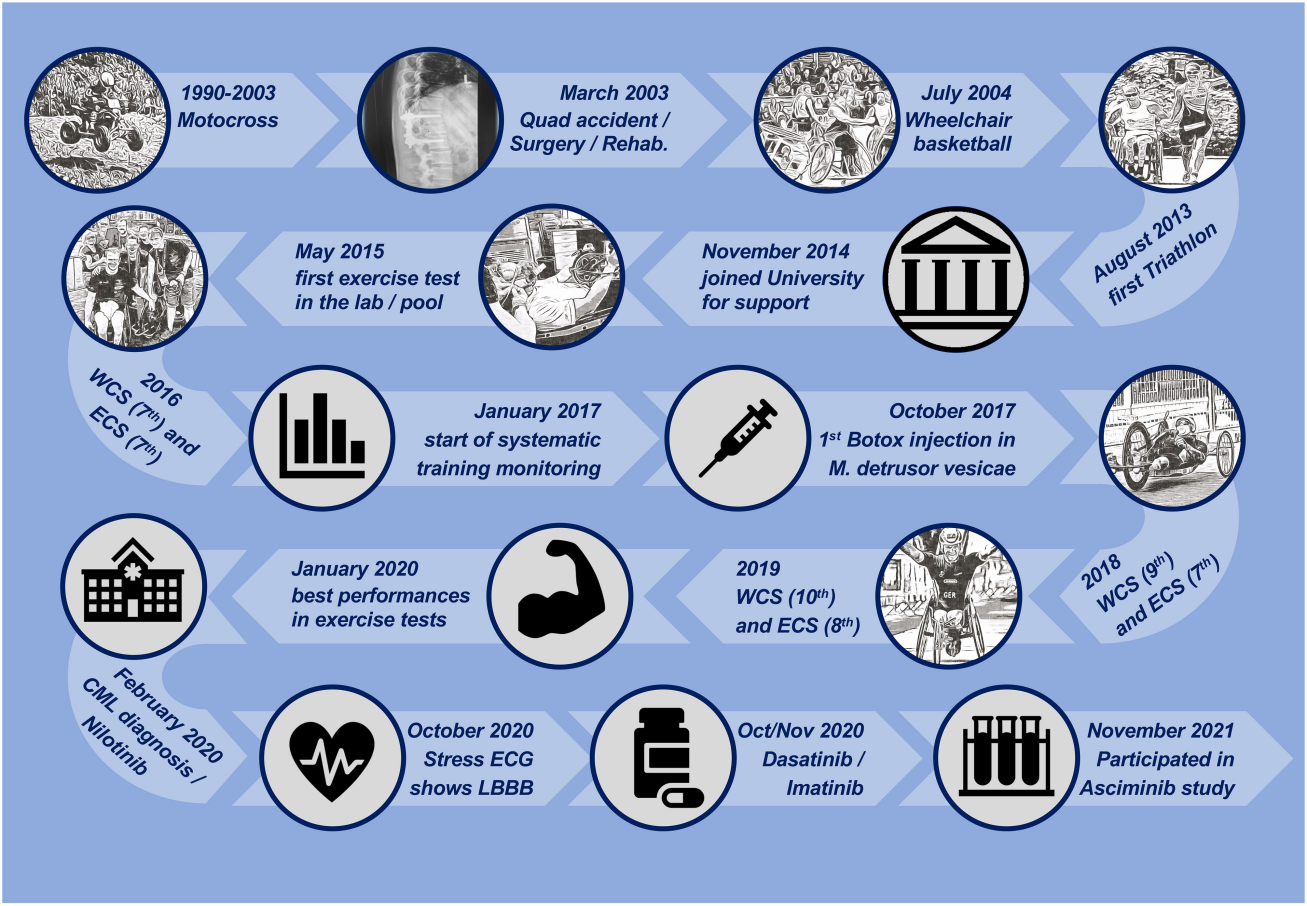
Timeline of the athlete's personal history and milestones during the exposure. CML, chronic myeloid leukemia; ECG, electrocardiogram; ECS, european championship; SCI, spinal cord injury; LBBB, left bundle branch block; WCS, world championship.

In November 2014, a 30-year-old male wheelchair athlete (ID: BL) with SCI classified as ASIA C (20) contacted our university and asked for support regarding his athletic orientation toward paratriathlon (Figure 1). BL had participated in professional wheelchair basketball for a decade and had already finished several triathlons including national championships. He wanted to have guidance in sport-specific training and testing to achieve his ultimate goal: participating in the Paralympic Games in either Rio de Janeiro (2016) or Tokyo (2020). The athlete gave written informed consent to take part in this study. Standardized guidelines for reporting were used (refer to Supplementary Material 1).

From 1990 to 2003, BL participated in motocross races. In March 2003, at the age of 18 years, he was involved in a quad bike accident that caused lumbar spine compression (ICD10-S32.01), kidney contusion (ICD10-S37.01), and incomplete paraplegia (ICD10-S34.71) with neurogenic bladder dysfunction (ICD10-N31.1) that required the permanent use of a wheelchair (ICD10-Z99.3). Immediate surgery stabilized the spine by internal fixation of the thoracolumbar junction. BL received physiotherapy, ergotherapy, and medical care during acute rehabilitation up to June 2004. He started wheelchair basketball soon after rehabilitation and played for several teams at the national level. Regular medical check-ups remained unsuspicious. In August 2013, he participated in his first sprint-distance paratriathlon.

## Methods

Due to the unique demands (focus on an upper extremity) of PTWC, we modified laboratory and field tests that are commonly performed by non-disabled athletes to assess BL's physical abilities in swimming, handcycling, and wheelchair racing. Depending on his performance in paratriathlon events (discipline-specific ranking and split times) and the outcomes of the exercise tests, the training prescription focused on disciplines and/or physiological parameters that seemed to provide particular performance gains. As such, handcycling was identified as the discipline with the highest potential for improvement. Moreover, the applied training concept was oriented toward similar case studies and the international literature that highlighted the value of high training volumes performed at low intensity (5, 6, 8, 21). In our study, low intensity is oriented toward maximal fat oxidation rate (Fat_max_) (22). Training camps of a 2-weeks duration were performed 2–3 times a year during the preparation period (typically between October and March). Procedures to systematically quantify his training in every discipline were applied (23).

### Exercise Testing

Prior to any testing, the participant received a medical check-up following the guidelines of the European Society of Cardiology, which includes the individuals' own medical, family, and personal history, a physical examination, and a resting electrocardiogram (24). The frequency of exercise tests increased from once per year in 2015–2016 (performed in May) to 4 times per year in 2018–2019 (performed at the beginning of the preparation and competition period as well as preceding the training camps). Also, the number of procedures increased over the years and finally required one testing day for swimming, handcycling, and wheelchair racing, respectively. Test days were separated by 1–2 rest days with (at most) low-intensity training at (Fat_max_). Besides rather common procedures in exercise testing which target maximal oxygen uptake (V°O_2max_) and/or lactate threshold (25), we developed procedures to determine maximal lactate accumulation rate (${\dot{\text{V}}\text{La}}_{\max}$) as a measure of the glycolytic metabolism. These procedures demonstrated sufficient reliability, were associated to physical performance in handcycling (26, 27) and running (28, 29), and were modified for this case study accordingly. ${\dot{\text{V}}\text{La}}_{\max}$ is derived from short sprint tests and calculated by dividing the increase in postexercise blood lactate concentration (BLC) by the assumed lactic period (in this study 3 s) (30, 31). Discipline-specific procedures to determine ${\dot{\text{V}}\text{La}}_{\max}$ are described below.

The high number of tests was physically and mentally challenging for BL and led to a trade-off between testing and training. Testing required access to various sports facilities and was dependent on weather conditions and temperature for wheelchair racing field tests. For tests including exhaustion in the laboratory, a medical doctor was on on-call duty which complicated scheduling. Procedures focused on aerobic as well as anaerobic parameters to create a holistic physiological profile of the athlete in all disciplines (30, 32). Furthermore, we wanted to supplement the ease of field testing with the standardization and reliability of lab tests.

In swimming, critical swim speed (CSS) was determined by performing 200-m and 400-m time trials in a 50-m pool (33). Later, testing was expanded by an initial 25-m sprint test and a closing 750-m time trial. The 750-m trial was requested and consequently performed by the athlete since this is the swimming distance in standard paratriathlon events and approximates his performance in competition. Immediately before and after the time trials, blood samples were collected from the earlobe and analyzed using an enzymatic-amperometric sensor chip system (Biosen C-Line, EKF-diagnostic GmbH, Barleben, Germany) to assess the net lactate production. Postexercise BLC of the 25-m sprint test was recorded every minute for 10 min to estimate ${\dot{\text{V}}\text{La}}_{\max}$.

In handcycling, an incremental test on an ergometer (Cyclus 2, RMB electronic automation GmbH, Leipzig, Germany) was performed to determine the power corresponding to a BLC of 4 mmol·l^−1^ (P_OBLA_) (25) and the peak oxygen uptake (VO_2peak_) which was measured by a spirograph (ZAN 600, nSpire Health, Inc., Longmont, CO, United States), as these parameters are significantly associated with handcycling performance (34–36). The incremental test started with an initial load of 20 W and increased intensity by 20 W every 5 min until the athlete attained subjective exhaustion (26). Furthermore, an isokinetic 15-s all-out sprint test was performed on the same ergometer to determine ${\dot{\text{V}}\text{La}}_{\max}$ (26, 27). Since V°O_2peak_ may depend on the used protocol (37, 38), an additional ramp test (80 W, 5 W, 15 s) was performed on some occasions to determine V°O_2max_, which “is defined as the highest rate at which oxygen can be taken up and utilized by the body during severe exercise” (39).

In wheelchair racing, an initial 110-m sprint test on an outdoor track was performed to calculate ${\dot{\text{V}}\text{La}}_{\max}$ analogously to previous studies in running (28). Later, time trials over 1,500 and 3,000 m were applied to determine performance, critical velocity (CV), and immediate post-exercise BLC. These trials were used to determine discipline-specific performance and use CV as an indicator of the high-intensity domain (40).

### Training Monitoring

The training was quantified by methods already applied in conventional triathlon (23). External (e.g., time, velocity, power, and cadence) and internal (e.g., heart rate) training measures were recorded by a sports watch or bike computer that was (even in swimming) connected with a heart rate monitor (Forerunner 920XT, Edge 20, HRM-Tri and HRM-Swim, Garmin International, Inc., Olathe, KS, United States). Although all these measures were used to schedule the training, heart rate was found to be most suitable for quantifying the training and comparing between the disciplines. Discipline-specific heart rate intensity zones (T5-T1) were determined as percentages of maximum heart rate with thresholds of 93, 85, 75, and 60%, respectively. These thresholds were found to fit well with training zones from physiological exercise testing (21) and attain stable results. Despite differences in power over time, the heart rate corresponding to a BLC of 2 and 4 mmol·l^−1^ was always 131 ± 1 and 158 ± 1 bpm which corresponded to ~70 and ~80% maximal heart rate, respectively. Although the maximal heart rate was 179 bpm in swimming, handcycling and wheelchair racing attained values up to 188 bpm. Training load (TL) was quantified by several parameters to assess their comparability. The training impulse of a five-zone model (TRIMP_5_) was calculated by multiplying the minutes spent in each zone by their identifier (23). For a three-zone model (TRIMP_3_), the highest (T4–T5) and lowest zones (T1–T2) were combined and multiplied analogously by 1–3 (41). The polarization index was calculated according to the literature (42). Besides these scientific procedures of quantification, the athlete's sensation of acute fatigue (“heavy arms”) was subjectively recorded. This type of sensation indicates that a typical feeling of soreness following training is exceeded and may affect the following training sessions.

As a subjective measure of TL, session ratings of perceived exertion (sRPE) were recorded (43). The total load index (TLI) was calculated by multiplying training duration (min) and sRPE. Training sessions were synchronized *via* the Garmin-Connect-App and entered in an EXCEL spreadsheet to calculate TL for a whole Paralympic cycle (2017–2020). Data are expressed as a sum of 4-weeks blocks.

### Strength Training and Physiotherapy

Additional strength training and physiotherapy were assumed to be crucial for meeting the high demands on the upper extremities in PTWC, improving performance, and minimizing the risk of overuse injuries (14, 44, 45). Every strength training session was preceded by a movement preparation that included stretching and activating exercises for the upper extremity and trunk. Additionally, exercises targeting the external shoulder rotators were performed with elastic bands to improve stability and avoid muscular imbalance (46).

Stationary strength training was performed on automatically guided and software-controlled devices (Milon Industries GmbH, Emersacker, Germany) that monitored the eccentric and concentric loads of rowing, bench press, trunk flexion/extension, and pull-down exercises. Concentric failure was attained after a desired number of reps (±2) that decreased during the preparation period (starting annually in October/November). After 4–8 weeks of 2 × 20 reps (30 s rest) and 3–6 weeks of 2 × 12 reps (45 s rest), a high-intensity block of 4 × 6 reps (70 s rest) was applied for 2 weeks. Maintenance (moderate) training once a week was applied during the rest of the year. Since *M. deltoideus, M. biceps* brachii, and *M. trapezius* are highly activated in handcycling and are assumed to be major contributors to tonicity/discomfort (47), preventive manual therapy was applied 1–2 × per week.

### Changes in the Intervention

Training contents and periodization were largely influenced by the athlete's work duration, the access to sports facilities (e.g., swimming pool), the short-term announcements of paratriathlon starting lists, and perceived discomfort/fatigue. Therefore, flexibility and trade-offs were common practices during the intervention. In June/July 2017, severe physical complaints caused by neurogenic bladder dysfunction led to a mandatory break in training. Consequently, *M. detrusor vesicae* were inhibited by annual injections of Botulinum toxin (Botox®). During a training camp in August 2019, BL attained a stress fracture of two of his ribs (ICD 10-S22.42, 6th and 7th), which resulted in a reduced TL for several weeks and rescheduling of international paratriathlon events. In 2020, the coronavirus pandemic caused several restrictions like the first lockdown (in Germany from April to May) or the closure of sports facilities that significantly affected psychological variables in amateur and recreational athletes (48). Although this also applied to BL, CML diagnosis and treatment overshadowed pandemic effects. Accordingly, vigorous training especially at high intensity had to be avoided for several months.

### Chronic Myeloid Leukemia

The most crucial challenge of this exposure was caused in February 2020. A training camp on Lanzarote had to be canceled after a few days due to spontaneous and sustained nausea, discomfort, and remarkably reduced physical performance. Initial white blood cell differentiation showed elevated myelocytes (9.0%), metamyelocytes (3%), and promyelocytes (2%). On 14 February, BCR-ABL1 transcripts (Type e13a2) of 56.8% confirmed CML (ICD10-C92.1). BL started treatment right away with the second generation TKI Nilotinib (Tasigna, 150 mg, 2-0-2). In the following months, BL started experiencing thoracic pain—especially during exercise. In October 2020, an electrocardiogram during incremental handcycling exercise demonstrated an exercise-induced left bundle branch block (ICD10-I44.7). Since this was interpreted as a side effect of Nilotinib, BL continued treatment with Dasatinib (Sprycel, 100 mg, 1-0-0). However, due to intense headache and vertigo, treatment was continued with Imatinib (Glivec, 400 mg, 1-0-0) and prescribed from November 2020 onward. In July 2021, the Imatinib dosage was reduced to 300 mg due to gastrointestinal complaints and increasing anemia (Hemoglobin toward 13 g/day). In November 2021, BL started participating in a clinical study that examines the effects of Asciminib medication (40 mg/day, 1-0-1) on BCR-ABL1 development and side effects in patients who have previously been treated with ≥2 ATP-binding site TKIs. The development of BCR-ABL1 levels over time is illustrated in Supplementary Material 2.

## Results

### Physical Exercise Tests

**Figure 2:**
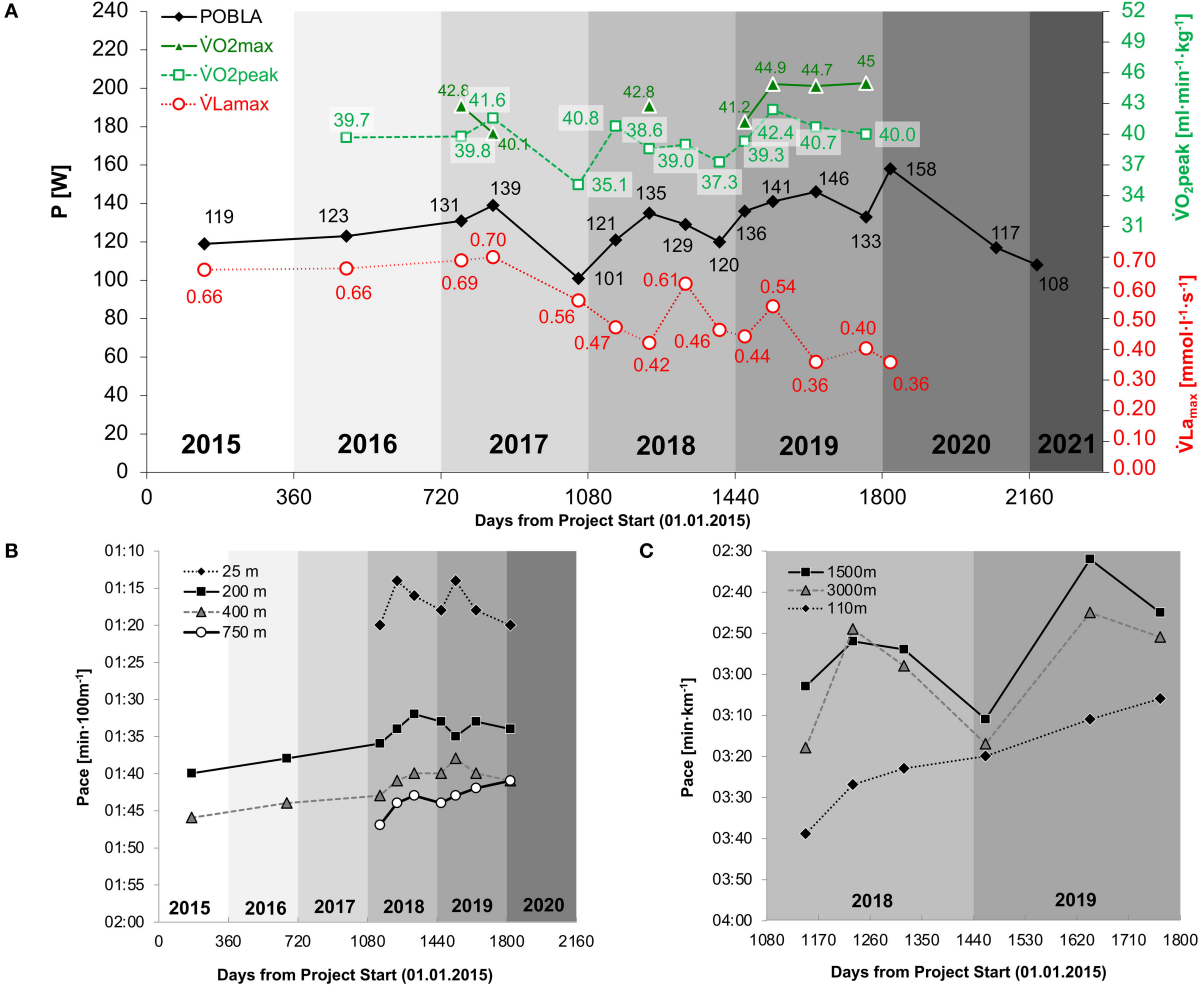
Performance development in handcycling **(A)**, swimming **(B)**, and wheelchair racing **(C)** ${\dot{\text{V}}\text{La}}_{\max}$, maximal lactate accumulation rate (mmol·l^−1^·s^−1^); V°O_2max_, maximal oxygen uptake (ml·min^−1^·kg^−1^); V°O_2peak_, peak oxygen uptake (ml·min^−1^·kg^−1^).

In accordance with his ranking at international paratriathlon events (Figure 1), exercise tests demonstrated an increase in physical performance. In handcycling, *P*_OBLA_ increased from 101 W in 2017 to 158 W in 2020 (Figure 2A). In this period, ${\dot{\text{V}}\text{La}}_{\max}$ decreased from 0.56 to 0.36 mmol·l^−1^·s^−1^. V°O_2peak_ showed the highest annual values following the preparation period and tended to decrease during the competition (March to July/August) and the transition period (August/September to October). Figure 2B illustrates performance development in swimming. From 2015 to 2020, CSS increased from 1:34 to 1:27 min·100 m^−1^. From 2018 to 2020, the sport-specific 750-m pace improved from 1:47 to 1:41 min·100 m^−1^ while the sprint pace demonstrated an annual pattern. In wheelchair racing, CV increased from 2:55 min·km^−1^ in June 2018 to 2:19 in July 2019 (Figure 2C). Overall, postexercise BLC was found to be highest in swimming and lowest in wheelchair racing.

### TL and Training Intensity Distribution

**Figure 3:**
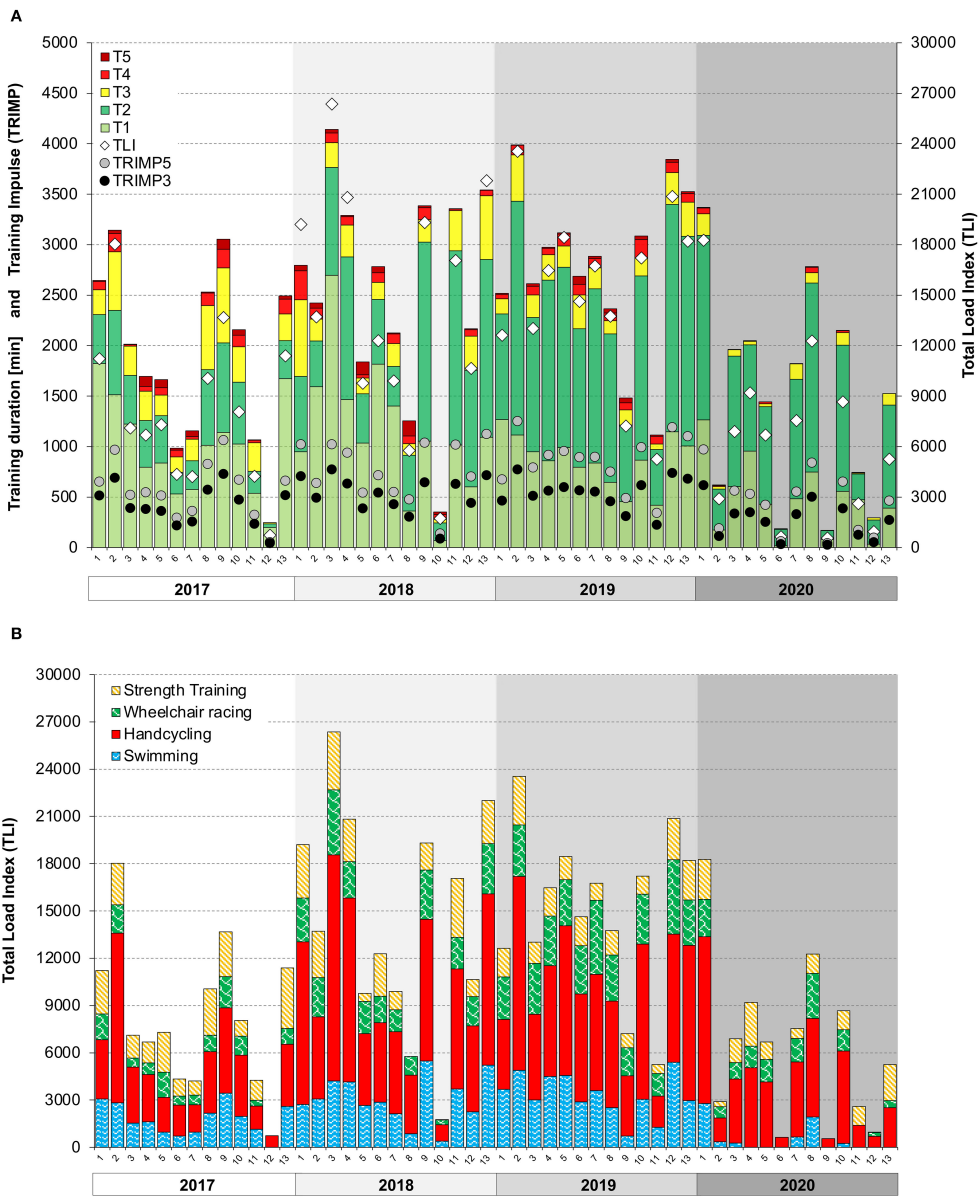
Training monitoring from 2017 to 2020. **(A)** Total training duration in every exercise zone (T1–T5) over time in 4-week blocks (13 for each year). TRIMP and total load index (TLI) over time in 4-week blocks (13 for each year). (TRIMP_3_), training impulse based on a three-zone model; TRIMP_5_, training impulse based on a five-zone model; TLI, total load index; sRPE × training duration [min]. **(B)** TLI in every discipline over time in 4-week blocks (13 for each year). TLI, total load index; sRPE × training duration (min).

Annual training duration increased from 414 h in 2017 to 604 h in 2019 (Table 1). In this period, the proportion of handcycling and wheelchair racing increased, while swimming was maintained and strength training was decreased. BL reduced his office work by 50% in 2018 and started full-time training in 2019. The distribution of TL between disciplines was similar for all measures. In 2020, the annual training duration decreased to 317 h and demonstrated a relatively high proportion of handcycling and strength training and a considerably low amount of swimming. Figure 3A illustrates overall TL and time spent in various intensity zones from 2017 to 2020. The least variation in overall TL occurred in 2019. TLI distribution among disciplines over time is illustrated in Figure 3B. Periods of increased training volume seemed to primarily result from handcycling exercise, whereas the strength TL was high during preparation and low during the competition period. During training camps, daily TLI and TRIMP_3_ were found to be around 2,000 and 350, respectively, and were separated by rest days on a 2:1 to 3:1 basis. An example training camp from February 2019 is illustrated in Supplementary Material 3. Weekly training duration during the camps attained values of 20–30 h with an overall TID of ~ (84-13-3%). TLI, TRIMP_3_, and TRIMP_5_ demonstrated high correlations on a weekly (*R*^2^ = 92-98%) and monthly (*R*^2^ = 90–97%) basis, with the highest correlation between TRIMP_3_ and TRIMP_5_ (refer to Supplementary Material 4). Sensations of acute fatigue (“heavy arms”) were frequently reported (~once per month) and were highest during a high-intensity block periodization (December 2018).

**Table 1:** Yearly and discipline-specific training load (TL) during the Olympiad.

**Parameter**	**Discipline**	**2017**	**2018**	**2019**	**2020**
Duration (h)	Overall	414	557	604	317
	Swimming	83 (20%)	110 (20%)	136 (22%)	24 (8%)
	Handcycling	161 (39%)	258 (46%)	271 (45%)	182 (57%)
	Wheelchair racing	54 (13%)	82 (15%)	116 (19%)	46 (15%)
	Strength training	91 (22%)	97 (17%)	79 (13%)	59 (19%)
TRIMP_3_	Overall	32,279	40,744	42,289	20,406
	Swimming	6,832 (21%)	8,670 (21%)	9,650 (23%)	1,556 (8%)
	Handcycling	12,266 (38%)	18,238 (45%)	18,830 (45%)	11,200 (55%)
	Wheelchair racing	4,715 (15%)	6,643 (16%)	8,909 (21%)	3,215 (16%)
	Strength training	5,573 (17%)	5,898 (14%)	4,783 (11%)	3,526 (17%)
TRIMP_5_	Overall	44,731	59,604	67,487	33,337
	Swimming	9,778 (22%)	13,021 (22%)	15,382 (23%)	2,567 (8%)
	Handcycling	17,486 (39%)	28,103 (47%)	31,910 (47%)	20,064 (60%)
	Wheelchair racing	6,896 (15%)	10,092 (17%)	14,820 (22%)	5,626 (17%)
	Strength training	6,229 (14%)	6,445 (11%)	5,200 (8%)	3,716 (11%)
TLI	Overall	107,083	188,383	198,002	82,423
	Swimming	22,951 (21%)	39,678 (21%)	43,059 (22%)	6,247 (8%)
	Handcycling	46,408 (43%)	94,084 (50%)	93,294 (47%)	48,028 (58%)
	Wheelchair racing	13,133 (12%)	28,486 (15%)	39,910 (20%)	13,355 (16%)
	Strength training	24,593 (23%)	26,348 (14%)	21,749 (11%)	14,794 (18%)

The TID showed a pyramidal periodization and a shift toward high volume training from 2017 (77-17-6%) to 2019 (88-8-4%) (refer to Supplementary Material 5). In 2020, overall TID was found to be 94-5-1%. The highest percentage of high-intensity was found in wheelchair racing (~10%), followed by swimming/handcycling (~5%) and strength training (<0.5%). Training intensity of stationary strength training demonstrated an increase during preparation periods as well as over the years (Supplementary Material 6).

### BCL-ABL1 Transcripts

From an initial value of 56.8 in February 2020, BCL-ABL1 transcripts decreased substantially after 41, 91, and 248 days to values of 5.65, 0.0416, and 0.008, respectively (Supplementary Material 2). Reduced BCL-ABL1 transcripts imply a reduced tumor load indicating that a patient is positively responding to the applied therapy.

## Discussion

This case report represents one of the most extensive descriptions of complex exercise testing and long-term training monitoring in Paralympic sports. We demonstrated that the physical abilities of a paratriathlete in the wheelchair category improved with increasing TL and reductions in office work duration. Training intensity distribution (TID) showed a pyramidal periodization and shift toward low-intensity training. However POBLA increased and ${\dot{\text{V}}\text{La}}_{\max}$ decreased over the years, V°O_2peak_ indicated an annual pattern and attained the highest values following the preparation period. With CML diagnosis and its treatment, TL (especially with respect to high intensities) and physical abilities substantially decreased. This hindered the athlete from competing in international events and ultimately from qualifying for the Paralympic Games in Tokyo.

A similar description was recently provided for a female paraswimmer during a Paralympic cycle (4). Although annual training hours and their development over the years were similar to this case study, the TID of the paraswimmer demonstrated an even higher percentage of low-intensity training. This is influenced by the fact that the authors used a session goal approach to determine TID, whereas a time-in-zone approach was used in this case study (49). When compared with previous studies in handcycling (5, 6), the performance gains were lower, and TID was less polarized in this study. However, these authors used time in power zones rather than heart rate zones to calculate TID, which was shown to differ between methods (50)—especially during high-intensity sessions (51). Future studies need to examine these differences in handcycling.

Training load was quantified by means of separate internal measures (TLI and TRIMP) in order to ensure comparability and standardization between paratriathlon disciplines. The high correlation between these parameters indicates an overall agreement between objective and subjective measures of TL. However, we noticed that the discrepancy between subjective and objective TL (Supplementary Material 3) coincided with acute fatigue (“heavy arms”), especially when subjective TL was substantially higher when compared with measures of objective TL. Thus, considering both types of loads and their discrepancy could be helpful for carefully monitoring daily TL and preventing acute fatigue and non-functional overreaching, especially during training camps. Despite the assumptions of previous research (52), recent findings indicate that ratios between subjective and objective measures (sRPE:TRIMP) do not provide additional information to monitor fatigue in cyclists (53). Furthermore, it was shown that TLI is not associated with alterations in physical capacity, whereas the time attained in heart rate zone 2 of 3 significantly correlated with improvements in V́O_2peak_ during the preparation for the HandbikeBattle (54). This is in line with the initially high V°O_2peak_ values (relative to his development) of BL (when a lot of zone 2 training was performed) and the fact that V°O_2peak_ only slightly increased with high-volume low-intensity training (2018–2019).

A similar TID as in this case study was reported for a male long-distance paratriathlete with below-the-knee-amputation (8). The increase in low-intensity percentages over time might be due to the mere increase in training volume and/or the fact that the BL became increasingly patient about performing his training sessions in the prescribed training zones. However, this might also indicate that the training prescription of BL is overly focused on high-volume rather than high-intensity training. In fact, we tried to apply high-intensity training in a block periodization, which has been shown to be an adequate training strategy to improve V°O_2max_ (55). However, the athlete did not tolerate more than three high-intensity interval sessions in a week due to acute fatigue. This might be due to the fact that wheelchair triathletes purely rely on their upper extremities during training and activities of daily living. Therefore, the overall higher load applied on the upper extremities increases the risk for acute fatigue, which is less severe in conventional triathletes. We experienced that the duration at a high intensity (heart rate zones 4 and 5) was higher and more easily triggered by performing wheelchair racing rather than handcycling. However, this discipline comes along with a substantially higher shoulder load (56). Due to the BL's medical complaints, the fluctuations of TL between training blocks were higher compared with previous studies (4, 6, 8), which affected training consistency, which is observed in “*full-time, year-round athletes”* (57).

In our case study, reducing employment increased the athlete's amount of available time and energy which allowed for more (intense) training. This increase in training duration is accompanied by an enhanced training adaptation as documented by the exercise tests. This increase in training and recovery duration is facilitated by corresponding sponsorship that was not constantly available for this athlete. Therefore, at first, training camps were tightly scheduled due to restricted training time. Although previous studies reported an average training volume during training camps of 137 ± 33% of preceding (regular) TL in the absence of acute fatigue or excessive stress (58), BL experienced a considerably higher load during the initial training camps (>200%) when he frequently reached his physical limits. From 2018 onward, we provided a less extensive and more flexible schedule in the following camps, performed daily (objective and subjective) monitoring, and implemented more (relaxing) rest days that substantially improved the feasibility, recovery, and mood. In future projects, the latter might be complemented by the Profile of Mood States Questionnaire that was successfully applied to wheelchair marathoners (7) and elite paratriathletes (58). To prevent acute fatigue and improve training quality during training camps, suitable strategies of micro-periodization were established. For example, combining high-intensity wheelchair racing in the morning followed by low-intensity handcycling was appropriate to properly exercise and recover on intensified days. Rest days consisted of an easy swim session and moderate strength training.

The characteristic pattern of VO_2peak_ development within each season (with the highest values observed after the preparation period) is similar to those reported in a world-class middle-distance runner (59). We assumed that the observed pattern shows a typical build-up, followed by a plateau and subsequent decline in performance during detraining. However, the athlete's paratriathlon performance in the competition was less affected over the respective years in terms of overall and split times. Jones demonstrated that world-class endurance performance can improve over several years despite a decrease/stagnation of V°O_2max_ as long as submaximal parameters improve (60, 61). In our case, improvements in maximal fat oxidation and/or movement economy might be the reason for the less severe decline in sport-specific performance. Especially during long rides, an improved “durability” in terms of an improved tolerance and less severe increase in heart rate during prolonged exercise were observed over the years which might be due to the high volumes of low-intensity training (62).

According to previous simulation approaches of glycolysis and oxidative phosphorylation, ${\dot{\text{V}}\text{La}}_{\max}$ and V°O_2max_ interact to determine lactate threshold (32). In simple terms, it is assumed that net lactate production results from the difference between the rate of lactate formation (as a percentage usage of ${\dot{\text{V}}\text{La}}_{\max}$) and the rate of lactate removal (which is assumed to be proportional to oxygen uptake). As such, maximal lactate steady state demonstrates the highest equilibrium of lactate formation and removal (net lactate production = 0) (63). This relationship is indicated by following the development of P_OBLA_ in Figure 2A. If we assume that P_OBLA_ is improved by an increase in V°O_2peak_ and/or decrease in ${\dot{\text{V}}\text{La}}_{\max}$ (and *vice versa*), we can qualitatively estimate the alterations in *P*_OBLA_. For example, *P*_OBLA_ decreased from 135 to 129 W despite a constant VO_2peak_ (probably) due to a huge increase in ${\dot{\text{V}}\text{La}}_{\max}$ immediately after a training camp. This is in line with a previous study highlighting that ${\dot{\text{V}}\text{La}}_{\max}$ significantly decreased after only 2 weeks of sprint interval training in trained cyclists (64). However, research on ${\dot{\text{V}}\text{La}}_{\max}$ adaptations is generally sparse. The reduced ${\dot{\text{V}}\text{La}}_{\max}$ values in our study are in accordance with previous research in ultra-endurance cycling, demonstrating a decrease in ${\dot{\text{V}}\text{La}}_{\max}$ during a prolonged period of high-volume low-intensity training (65). Since ${\dot{\text{V}}\text{La}}_{\max}$ was found to be increased by various forms of resistance exercise (66), an intensified fine-tuning of strength and sport-specific training contents was applied. Our preliminary findings demonstrated that ${\dot{\text{V}}\text{La}}_{\max}$ is affected by exercise modality, highest in swimming and lowest in wheelchair racing, which might be due to the usage of muscle mass. Although the reliability of ${\dot{\text{V}}\text{La}}_{\max}$ has been sufficiently assessed in handcycling (26, 27), future studies need to examine ${\dot{\text{V}}\text{La}}_{\max}$ in swimming and wheelchair racing.

There are several limitations of this case report that need to be mentioned. Given the high number of contextual variables (e.g., medical, logistical, nutritional, and social), training was frequently adapted to the acute circumstances, which makes it challenging to highlight causations. In PTWC triathlon, the need for various materials (handcycling and racing wheelchair), dependence on barrier-free facilities, and the pure focus on upper extremity locomotion demonstrate substantial constraints that the athletes have to overcome in order to be competitive. Especially as far as side effects of CML and its treatment are concerned, it is likely that experienced fatigue and reduced physical activity interact to ultimately decrease performance (17). Furthermore, TID is purely based on heart rate zones and as such hardly comparable with studies using session goals or time in power zone approaches.

In conclusion, this case report illustrates the training monitoring and performance development of a triathlete with SCI and CML during a Paralympic cycle. We demonstrated the need for careful training prescription in PTWC triathletes to improve performance in the absence of acute fatigue, overuse injuries, and non-functional overreaching. We encouraged athletes and coaches to refrain from overly extensive and/or intense training schedules and recommend the application of objective and subjective monitoring tools. This is stressed by the high demands on the upper extremities in wheelchair triathletes, who require special support, sponsors, and training prescriptions.

## Athlete Perspective

“In my opinion, having a great team of coaches and supporters was essential for gaining the last boost during the highs and lows of this journey. The same applies to those who gave medical assistance, which was highly important—especially in my case. Health complaints (e.g., bladder infections) kept me from performing in training and competitions, which was also mentally challenging. Accepting CML diagnosis and its consequences for fulfilling my goal took some time, even though my SCI-background helped to cope. I would like to share my experiences with sports and diseases to inspire others in the future.”

## Data Availability Statement

The original contributions presented in the study are included in the article/Supplementary Material, further inquiries can be directed to the corresponding author.

## Ethics Statement

Ethical review and approval was not required for the study on human participants in accordance with the local legislation and institutional requirements. The patients/participants provided their written informed consent to participate in this study. Written informed consent was obtained from the individual(s) for the publication of any potentially identifiable images or data included in this article.

## Author Contributions

OJQ planned the triathlon-specific training, participated in training camps, collected and analyzed the data, and drafted the manuscript. BL volunteered in this study, performed the training and exercise tests, and provided insights into his perspective. FL and PB were handlers at competitions and participated in training camps. FL provided logistical planning. PB planned and coached strength and conditioning contents and advised BL on aspects of nutrition and supplements. TF provided medical check-ups and supervised medical aspects of the project. All authors reviewed, edited, and approved the manuscript.

## Conflict of Interest

The authors declare that the research was conducted in the absence of any commercial or financial relationships that could be construed as a potential conflict of interest.

## Publisher's Note

All claims expressed in this article are solely those of the authors and do not necessarily represent those of their affiliated organizations, or those of the publisher, the editors and the reviewers. Any product that may be evaluated in this article, or claim that may be made by its manufacturer, is not guaranteed or endorsed by the publisher.

## Abbreviations

BLC: blood lactate concentration (mmol·l^−1^)
CML: chronic myeloid leukemia
CSS: critical swim speed
CV: critical velocity
P_OBLA_: power according to a lactate concentration of 4 mmol·l^−1^
PTWC: wheelchair category in paratriathlon
sRPE: session ratings of perceived exertion (scale from 1 to 10)
TKI: tyrosine kinase inhibitors
TLI: Total load index [sRPE × training duration (min)]
TRIMP_3_: training impulse (based on three-zone model)
TRIMP_5_: training impulse (based on five-zone model)
${\dot{\text{V}}\text{La}}_{\max}$: maximal lactate accumulation rate (mmol·l^−1^·s^−1^)
V°O_2max_: maximal oxygen uptake (ml·min^−1^·kg^−1^)
V°O_2peak_: peak oxygen uptake (ml·min^−1^·kg^−1^).
