# Supplementary material for: Case Report: Training Monitoring and Performance Development of a Triathlete With Spinal Cord Injury and Chronic Myeloid Leukemia During a Paralympic Cycle
Source: Front Rehabil Sci. 2022 Jun 30;3:867089. doi: 10.3389/fresc.2022.867089 (PMC9487515; doi:10.3389/fresc.2022.867089)
Supplement: Supplementary file 1 [file Data_Sheet_1.zip › Supplementary Material 1.PDF]

CARE case report guidelines

CARE Checklist of information to include when writing a case report

(cc) BY-NC-ND

☑

Topic	Item	Checklist item description	Reported on Line
**Title**	1	The diagnosis or intervention of primary focus followed by the words "case report"	✓ 1–2
**Key Words**	2	2 to 5 key words that identify diagnoses or interventions in this case report, including "case report"	✓ 12
**Abstract (no references)**	3a	Introduction: What is unique about this case and what does it add to the scientific literature?	✓ 15–23
	3b	Main symptoms and/or important clinical findings	✓ 21–28
	3c	The main diagnoses, therapeutic interventions, and outcomes	✓ 30–34
	3d	Conclusion—What is the main "take-away" lesson(s) from this case?	✓ 35–42
**Introduction**	4	One or two paragraphs summarizing why this case is unique (**may include references**)	✓ 44–52
**Patient Information**	5a	De-identified patient specific information.	✓ 84–87
	5b	Primary concerns and symptoms of the patient.	✓ 87–91
	5c	Medical, family, and psycho-social history including relevant genetic information	✓ 93–125
	5d	Relevant past interventions with outcomes	✓ " - "
**Clinical Findings**	6	Describe significant physical examination (PE) and important clinical findings.	✓ " - "
**Timeline**	7	Historical and current information from this episode of care organized as a timeline	✓ Figure 1 85/86
**Diagnostic Assessment**	8a	Diagnostic testing (such as PE, laboratory testing, imaging, surveys).	✓ ✓ 134–191
	8b	Diagnostic challenges (such as access to testing, financial, or cultural)	✓ 140–143
	8c	Diagnosis (including other diagnoses considered)	✓ 94–114
	8d	Prognosis (such as staging in oncology) where applicable	✓ 124–125
**Therapeutic Intervention**	9a	Types of therapeutic intervention (such as pharmacologic, surgical, preventive, self-care)	✓ 127–132
	9b	Administration of therapeutic intervention (such as dosage, strength, duration)	✓ 217–236
	9c	Changes in therapeutic intervention (with rationale)	✓ 193–202
**Follow-up and Outcomes**	10a	Clinician and patient-assessed outcomes (if available)	✓ 206–215
	10b	Important follow-up diagnostic and other test results	✓ 112–114, 236
	10c	Intervention adherence and tolerability (How was this assessed?)	✓ 171–175
	10d	Adverse and unanticipated events	✓ 193–202
**Discussion**	11a	A scientific discussion of the strengths AND limitations associated with this case report	✓ 238–250
	11b	Discussion of the relevant medical literature **with references**.	✓ 251–263
	11c	The scientific rationale for any conclusions (including assessment of possible causes)	✓ 258–241
	11d	The primary "take-away" lessons of this case report (without references) in a one paragraph conclusion	✓ 264–269
**Patient Perspective**	12	The patient should share their perspective in one to two paragraphs on the treatment(s) they received	✓ 271–277
**Informed Consent**	13	Did the patient give informed consent? Please provide if requested	Yes ☑ No ☐
