# Supplementary material for: Case Report: Training Monitoring and Performance Development of a Triathlete With Spinal Cord Injury and Chronic Myeloid Leukemia During a Paralympic Cycle
Source: Front Rehabil Sci. 2022 Jun 30;3:867089. doi: 10.3389/fresc.2022.867089 (PMC9487515; doi:10.3389/fresc.2022.867089)
Supplement: Supplementary file 1 [file Data_Sheet_1.zip › Supplementary Material 5.DOCX]

| **Discipline** | **Parameter** | **2017** | **2018** | **2019** | **2020** |
| --- | --- | --- | --- | --- | --- |
| Overall | T1 + T2 [h] | 316.6 (76.5%) | 464.8 (83.4%) | 527.5 (87.4%) | 300.2 (94.2%) |
|  | T3 [h] | 71.1 (17.2%) | 63.9 (11.5%) | 50.1 (8.3%) | 15.1 (4.7%) |
|  | T4 + T5 [h] | 26.4 (6.4%) | 28.8 (5.2%) | 25.7 (4.3%) | 3.2 (1%) |
|  | PI | 1.45 | 1.58 | 1.66 | 1.30 |
|  |  |  |  |  |  |
| Swimming | T1 + T2 [h] | 59.6 (71.7%) | 82.2 (74.9%) | 114.1 (84.2%) | 22.2 (92.3%) |
|  | T3 [h] | 16.5 (19.9%) | 20.3 (18.5%) | 17.6 (13%) | 1.8 (7.6%) |
|  | T4 + T5 [h] | 7 (8.5%) | 7.2 (6.6%) | 3.8 (2.8%) | 0 (0.2%) |
|  | PI | 1.40 | 1.43 | 1.26 | 0.39 |
| Handcycling | T1 + T2 [h] | 131 (81.4%) | 224.3 (87.1%) | 240.9 (88.9%) | 178.5 (98.2%) |
|  | T3 [h] | 16.5 (10.3%) | 20.3 (7.9%) | 17.6 (6.5%) | 1.8 (1%) |
|  | T4 + T5 [h] | 13.5 (8.4%) | 13 (5%) | 12.6 (4.6%) | 1.5 (0.8%) |
|  |  | 1.82 | 1.74 | 1.80 | 1.90 |
| Wheelchair racing | T1 + T2 [h] | 36 (66.2%) | 62 (75.5%) | 93.5 (80.4%) | 40.9 (88.1%) |
|  | T3 [h] | 12.6 (23.2%) | 11.5 (14.1%) | 13.5 (11.6%) | 3.8 (8.3%) |
|  | T4 + T5 [h] | 5.8 (10.7%) | 8.5 (10.4%) | 9.3 (8%) | 1.7 (3.6%) |
|  |  | 1.48 | 1.75 | 1.74 | 1.58 |
| Strength training | T1 + T2 [h] | 89.9 (98.4%) | 96.3 (99%) | 79 (99.6%) | 58.7 (99.9%) |
|  | T3 [h] | 1.3 (1.4%) | 0.9 (1%) | 0.3 (0.4%) | 0.1 (0.1%) |
|  | T4 + T5 [h] | 0.1 (0.1%) | 0.1 (0.1%) | 0 (0%) | 0 (0%) |
|  |  | 0.85 | 1.00 | 0.00 | 0.00 |

**Supplementary Material 5.** Yearly overall and discipline-specific time intensity zones (T1-T3) and training intensity distribution (TID). PI = polarization index.
