# Supplementary figures and images for: Case Report: Training Monitoring and Performance Development of a Triathlete With Spinal Cord Injury and Chronic Myeloid Leukemia During a Paralympic Cycle
Source: Front Rehabil Sci. 2022 Jun 30;3:867089. doi: 10.3389/fresc.2022.867089 (PMC9487515; doi:10.3389/fresc.2022.867089)

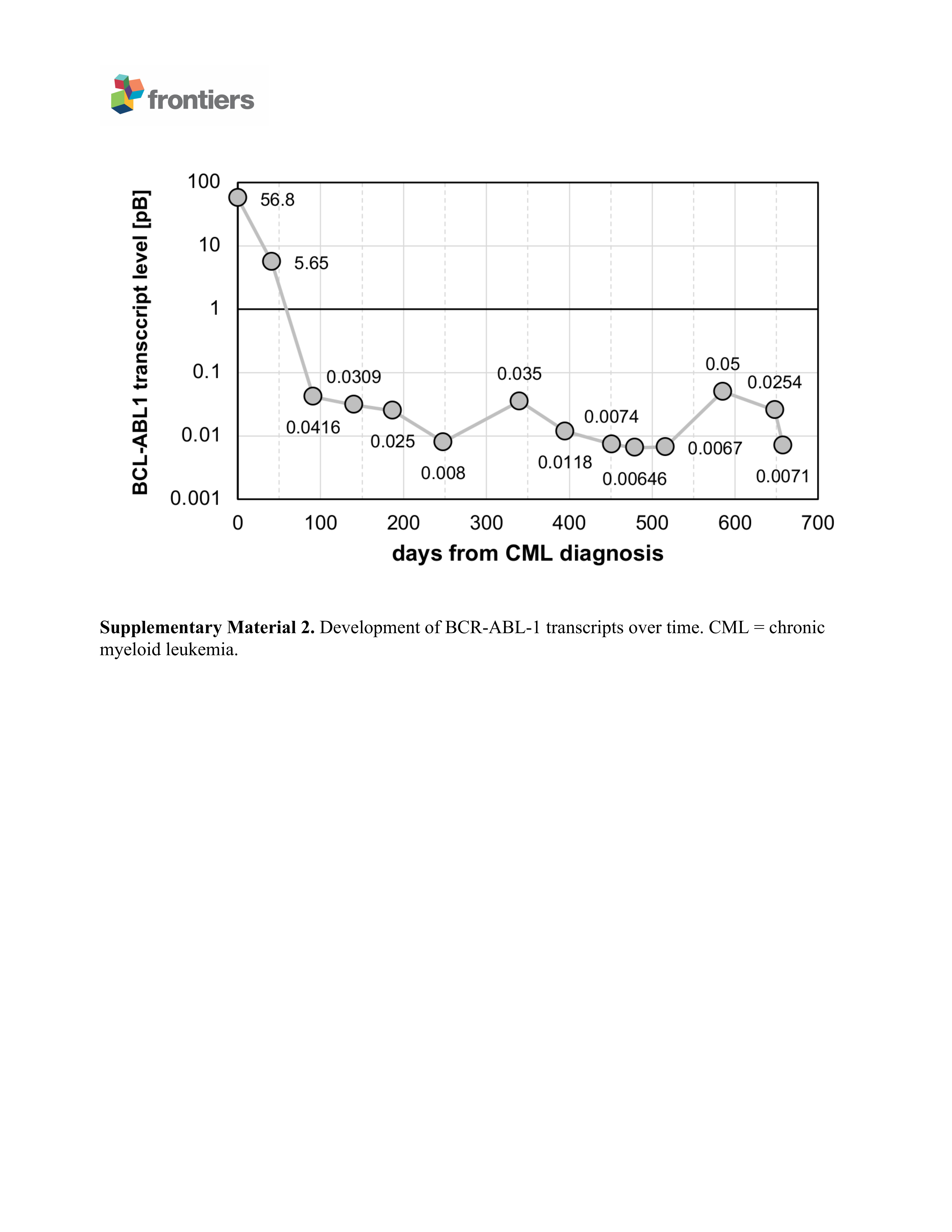

Supplement: Supplementary file 1 [file Data_Sheet_1.zip › Supplementary Material 2.JPEG]

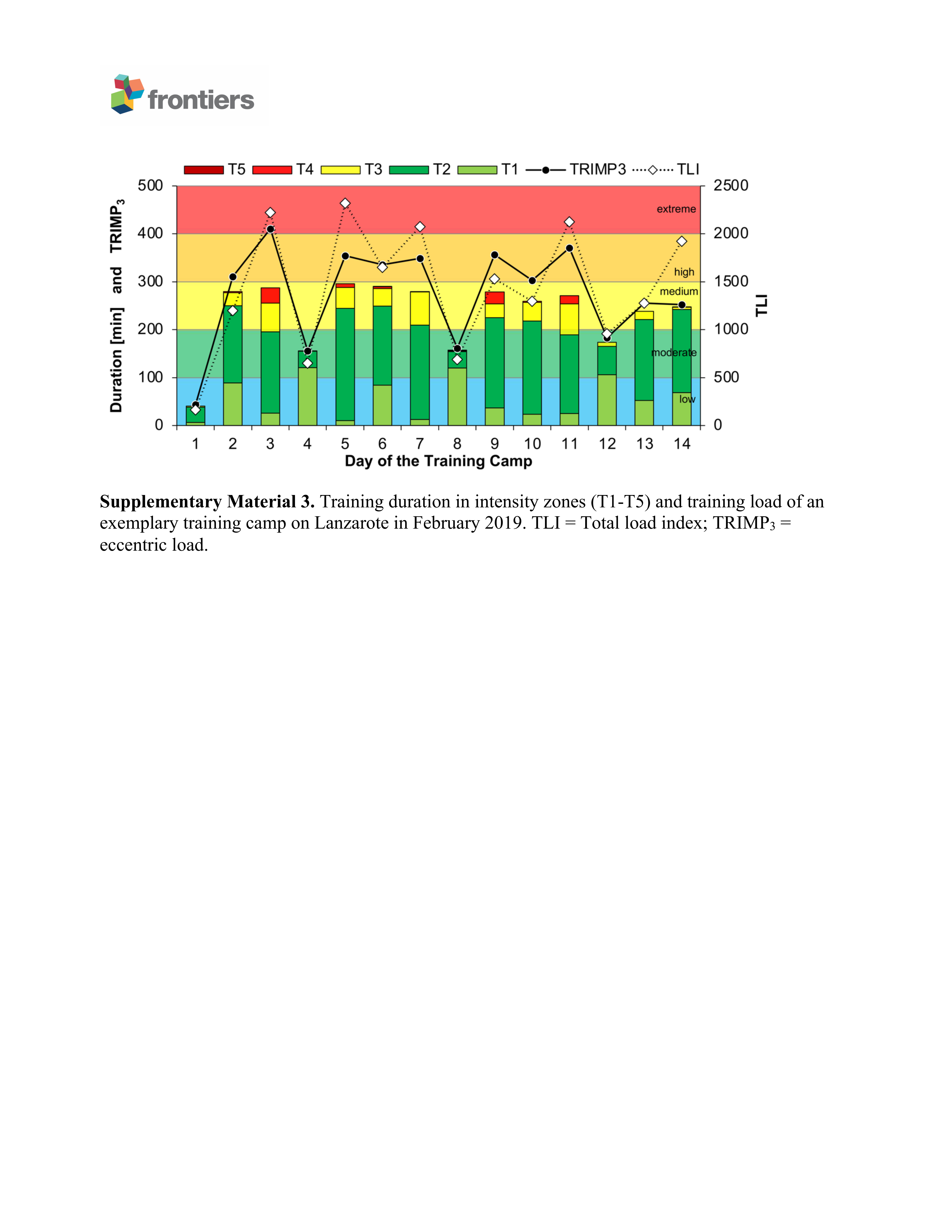

Supplement: Supplementary file 1 [file Data_Sheet_1.zip › Supplementary Material 3.JPEG]

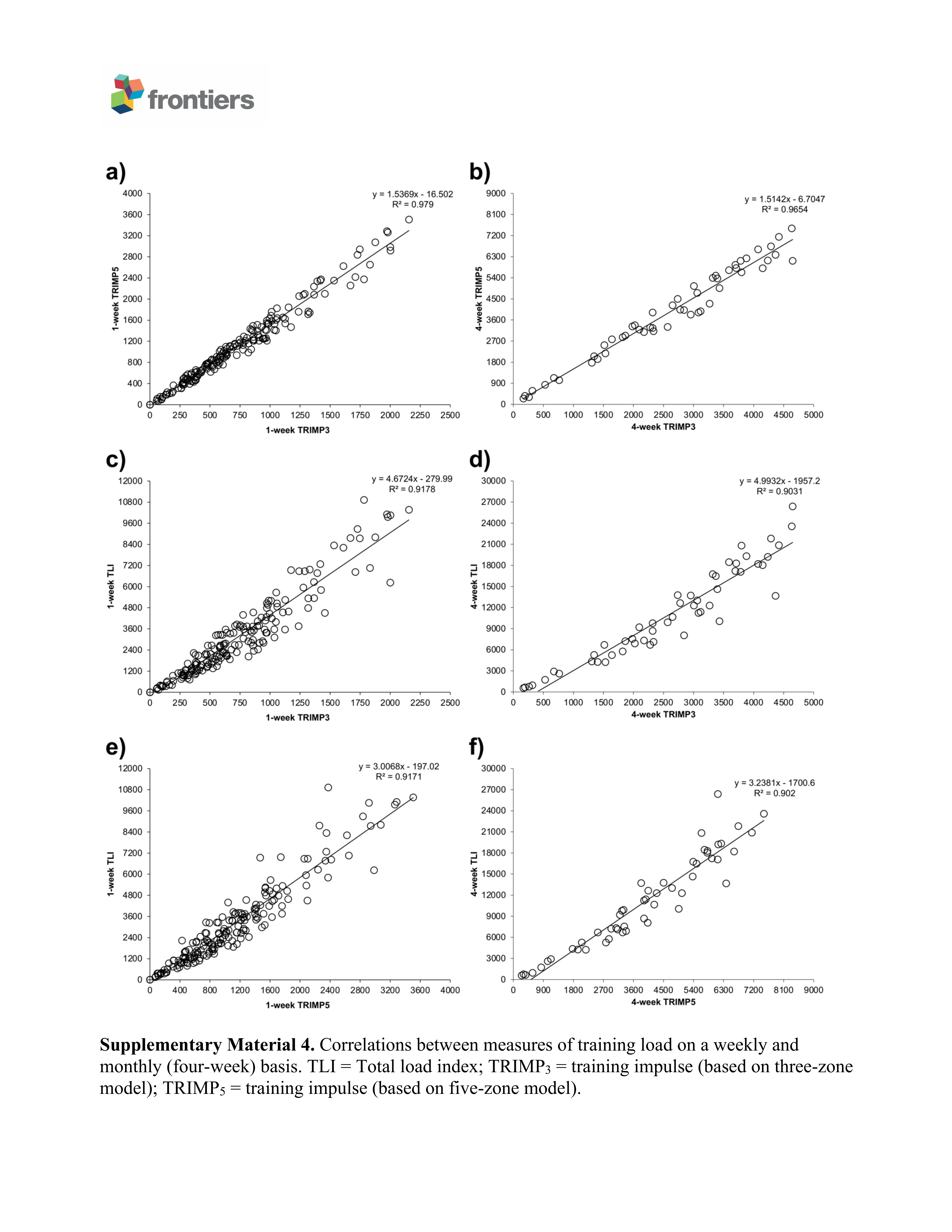

Supplement: Supplementary file 1 [file Data_Sheet_1.zip › Supplementary Material 4.JPEG]

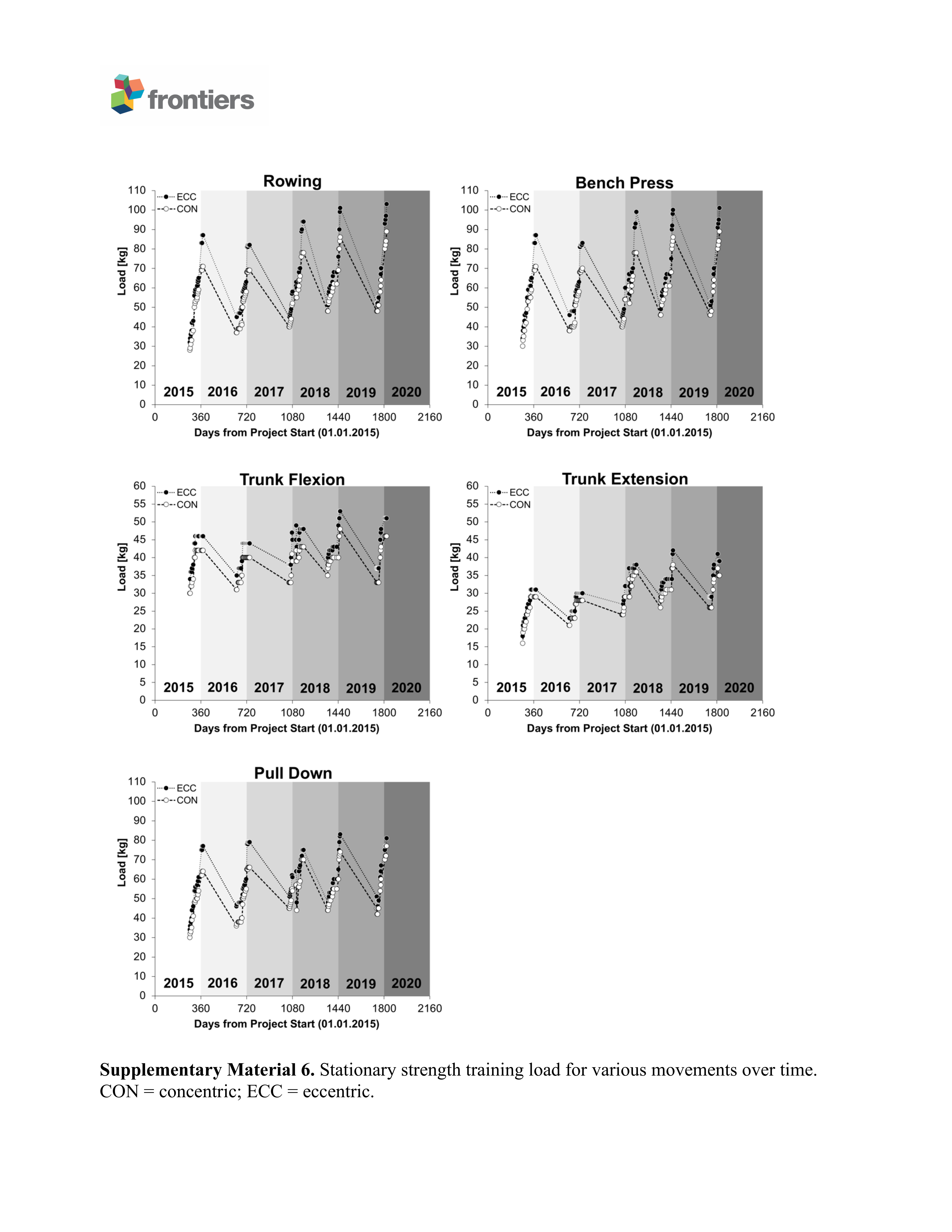

Supplement: Supplementary file 1 [file Data_Sheet_1.zip › Supplementary Material 6.JPEG]
